# Preclinical evaluation of cyclophosphamide and fludarabine combined with CD19 CAR-T in the treatment of B-cell hematologic malignancies *in vivo*

**DOI:** 10.32604/or.2024.049792

**Published:** 2024-05-23

**Authors:** ZHIGANG XIA, MENGYAO TIAN, YUCAI CHENG, WENFANG YI, ZEFAN DU, TIANWEN LI, YUCHEN WEN, LINDI LI, YONG LIU, CHUN CHEN

**Affiliations:** 1 Pediatric Hematology Laboratory, Division of Hematology/Oncology, Department of Pediatrics, The Seventh Affiliated Hospital of Sun Yat-sen University, Shenzhen, 518107, China; 2 Scientific Research Center, The Seventh Affiliated Hospital of Sun Yat-sen University, Shenzhen, 518107, China

**Keywords:** CD19 CAR-T, B-cell hematologic malignancies, Metabolism, *In vivo*

## Abstract

**Background:**

Chimeric antigen receptor T (CAR-T) cell therapy has achieved marked therapeutic success in ameliorating hematological malignancies. However, there is an extant void in the clinical guidelines concerning the most effective chemotherapy regimen prior to chimeric antigen receptor T (CAR-T) cell therapy, as well as the optimal timing for CAR-T cell infusion post-chemotherapy.

**Materials and Methods:**

We employed cell-derived tumor xenograft (CDX) murine models to delineate the optimal pre-conditioning chemotherapy regimen and timing for CAR-T cell treatment. Furthermore, transcriptome sequencing was implemented to identify the therapeutic targets and elucidate the underlying mechanisms governing the treatment regimen.

**Results:**

Our preclinical *in vivo* evaluation determined that a combination of cyclophosphamide and fludarabine, followed by the infusion of CD19 CAR-T cells five days subsequent to the chemotherapy, exerts the most efficacious therapeutic effect in B-cell hematological malignancies. Concurrently, RNA-seq data indicated that the therapeutic efficacy predominantly perturbs tumor cell metabolism, primarily through the inhibition of key mitochondrial targets, such as C-Jun Kinase enzyme (C-JUN).

**Conclusion:**

In summary, the present study offers critical clinical guidance and serves as an authoritative reference for the deployment of CD19 CAR-T cell therapy in the treatment of B-cell hematological malignancies.

## Introduction

B-cell malignancies represent a diverse array of hematologic neoplasms arising from various stages of B-cell differentiation. This heterogeneity encompasses a spectrum of lymphoproliferative disorders, notably B-cell lymphoma, B-cell leukemia, and plasma cell dyscrasias [1]. The recent decade has witnessed substantial advancements in the therapeutic modalities for B-cell malignancies, spurred by the introduction of novel agents like rituximab, Bruton’s tyrosine kinase (BTK) inhibitors, and groundbreaking innovations in immunotherapy, such as chimeric antigen receptor T cells (CAR-T). However, the pervasive challenges posed by drug resistance and recurrent disease episodes underscore the persisting clinical complexities associated with these malignancies.

The CAR-T therapeutic paradigm, a frontier in active immunotherapy, has burgeoned with a notable acceleration in recent years. This conceptual breakthrough can be attributed to Zelig Eshhar, who introduced the “chimeric antigen receptor (CAR)” paradigm in 1989, and was later brought to clinical relevance by Carl June in the successful treatment of a refractory and relapsed patient, Emily, in 2012 [2]. Concomitant advancements in genetic engineering have catalyzed the rapid progression of CAR-T clinical research in relapsed/refractory acute B-lymphoblastic leukemia, amplifying its therapeutic footprint [3]. Distinguished from traditional cellular immunotherapies by its specificity, CAR-T therapy heralds a new era in precision immunotherapy anchored on immune cells. The quintessential Chimeric Antigen Receptor (CAR) is structured with a single-chain variable fragment (scFv), a hinge region, a transmembrane domain, and an intracellular signaling activation domain. Notably, the scFv is an amalgamated protein embodying the variable regions of both the heavy and light chains of an immunoglobulin [4]. Employing either viral or non-viral vectors, CAR-T cells transfected with the aforementioned CAR structure recognize target antigens via a Major Histocompatibility Complex (MHC)-independent modus operandi, culminating in T cell activation and subsequent targeted cytotoxicity [5]. With the intrinsic limitations of autologous CAR-T cells, allogeneic or “universal” CAR-T cells are being envisaged as a pivotal evolutionary trajectory in this realm. A salient challenge tethered to universal CAR-T therapy is the potential for immune rejection by the host, coupled with unintended cytotoxicity against host tissues. To circumvent these impediments, strategies like the abrogation of the T-cell receptor (TCR) and human leukocyte antigen (HLA) on CAR-T cells have been postulated, with the aim of obliterating the immunogenicity of allogeneic T cells. Concurrently, engineering an optimal immune milieu within the host, often via preconditioning chemotherapy regimens, is postulated as an effective approach to mitigate immune rejection [6]. Nevertheless, a lacuna persists in the establishment of a definitive protocol delineating the optimal chemotherapy regimen and the temporal window for CAR-T cell administration post-chemotherapy. This accentuates the imperativeness of delving into preclinical explorations to decipher the ideal chemotherapy preconditioning landscape prior to CAR-T therapy and the subsequent timing of its administration.

In our quest to delineate the most efficacious chemotherapy preconditioning paradigm preceding CAR-T intervention and the optimal post-chemotherapy CAR-T therapeutic window, meticulous preclinical *in vivo* assessments illuminated that, in the context of B-cell hematological malignancies, CD19 CAR-T cell intervention manifested peak therapeutic efficacy following a five-day preconditioning regimen with cyclophosphamide and fludarabine. Subsequent investigations divulged that the primary mechanism underpinning this therapeutic prowess was the perturbation of tumor cell metabolism. This revelation carries profound implications, auguring well for the enhancement of CAR-T therapeutic outcomes and curtailing the recurrence rate of hematologic maladies in clinical settings.

## Materials and Methods

### Reagents

Cyclophosphamide (CTX) and Fludarabine (FDR) were purchased from MCE (Monmouth Junction, NJ, USA). Antibodies hCD19-APC (Cat#: 392504), hCD45-FITC (Cat#: 304006), hCD24-PE (Cat#: 311105) and hCD52-PE (Cat#: 316005) were from BioLegend (San Diego, CA, USA).

### Cell culture

Nalm-6 and Raji cell lines, both procured from the American Type Culture Collection (ATCC), were cultured in RPMI medium (Invitrogen, Shanghai, China), enriched with 10% fetal bovine serum (Biological Industries, Kibbutz Beit Haemek, Israel) and an antibiotic combination of 100 units/mL penicillin and streptomycin. Concurrently, Human embryonic kidney 293 cells (Lenti-X-293T) (ATCC) were sustained in Dulbecco’s Modified Eagle’s Medium (Invitrogen), supplemented with 10% FBS and 100 IU/mL penicillin/streptomycin. Culturing conditions were set to 37°C with a humidified atmosphere consisting of 95% air and 5% CO_2_, following previously established protocols [7]. Periodic checks were conducted to ensure the cell cultures were devoid of mycoplasma contamination.

### Generation of universal CAR-T cells

Drawing from the architectural details of the CD19 CAR vector in existing literature [8,9], we engineered a second-generation CD19 scFv-based CAR construct incorporating the 4-1BB molecular transmembrane region, intracellular co-stimulatory domain, and CD3ζ signaling area. Subsequent steps included coating a lentivirus with the CD19 CAR gene, followed by its concentration and purification. T cells were isolated from healthy donor peripheral blood, and post-activation, these cells were transfected with lentiviral vectors containing CARs. The transfection efficiency of CD19-CARs was evaluated using flow cytometry, specifically gauging the ratio of CD19-his-Tag. All research procedures conformed to the ethical standards laid out in the Declaration of Helsinki. The research protocol garnered approval from the Medical Ethics Committee of the Seventh Affiliated Hospital of Sun Yat-sen University (Shenzhen, China) (approval number: YQ-C-2022-16-01). Comprehensive informed consent was procured from all subjects or their designated representatives prior to their enrolment.

### Establishment of cell lines expressing luciferase stably

For the generation of lentiviral constructs, pMSCV-luciferase was transiently transfected into 293 T cells utilizing the PIK lentivirus packing system, mediated by polyethyleneimine (Polysciences, Inc., Warrington, PA, USA). Post a period of 48 or 72 h, the supernatants containing the virus were collected, filtered through a 0.45 μm filter, and then used to transduce growing Nalm-6 and Raji cells (0.5 × 10^6^ cells/mL) by spinoculation (1,500 g, 90 min, 32°C), with dilution occurring in a complete medium supplemented with polybrene (4 μg/mL) [10]. Subsequently, these cell lines were subjected to incubation with G418 (200 μg/mL) for approximately 10 days to derive stable clones expressing luciferase.

### Murine model of B-cell hematological malignancies

Male NSG mice, aged between 6 to 8 weeks (procured from Gem Pharmatech Co., Ltd., Nanjing, China), were intravenously inoculated with Nalm-6-luc cells (5 × 10^5^ cells in 200 μL PBS) and Raji cells (5 × 10^6^ cells in 200 μL PBS) through their tail veins. The subsequent day, these mice were randomly allocated to either control or experimental groups, and accordingly administered with either a vehicle (PBS) or an experimental treatment. Bioluminescent imaging (BLI) was executed at intervals—on days 0, 7, 14, and 21 post-treatments—to monitor the cellular proliferation, as depicted by luciferase signals. Regular health assessments of the mice were performed. By day 21, all mice were euthanized, leading to the extraction of spleens and bone marrow. Mononuclear cells, subsequently isolated from the bone marrow, underwent flow cytometry evaluation to determine the percentage of CD45^+^CD19^+^CD24^+^/CD45^+^CD19^+^CD52^+^ cells [11]. The entire study was carried out under stringent pathogen-free conditions at the Sun Yat-sen University animal care facility, with the protocols being approved by the University’s Institutional Animal Care and Use Committee (approval number: SYSU-IACUC-2021-000450).

### Flow cytometry analysis

Bone marrow or spleen-derived mononuclear cells were isolated following established protocols [12]. These cells were subsequently subjected to staining with antibodies: anti-CD45-FITC, anti-CD19-APC, and a combination of anti-CD24-PE and anti-CD52-PE. Following staining, flow cytometry was employed to determine the percentage of CD45^+^CD19^+^CD24^+^ and CD45^+^CD19^+^CD52^+^ malignant cell populations.

### RNA extraction, library construction, sequencing, and analysis

The procedure for RNA sequencing (RNA-seq) adhered to previously established methodologies [13]. In essence, mononuclear cells were isolated from the bone marrow, followed by discarding the supernatant and a subsequent washing step using PBS. Lysis was then achieved using Trizol reagent. Post total RNA extraction, oligo (dT) beads were employed to enrich for mRNA. This mRNA was subsequently fragmented using fragmentation buffer and reverse transcribed to cDNA employing the NEBNext Ultra RNA Library Prep Kit for Illumina (NEB #7530, New England Biolabs, Ipswich, MA, USA). Following purification, the cDNA fragment ends underwent repair, base addition, and ligation to Illumina sequencing adapters. Post-ligation, purification was achieved using AMPure XP Beads (1.0X), followed by polymerase chain reaction (PCR) amplification. The resulting cDNA libraries were sequenced by Gene Denovo Biotechnology (Guangzhou, China) using Illumina Novaseq6000.

### IHC and H&E staining assay

Mice were humanely euthanized, their spleens extracted, and the tissues were then fixed using formalin, embedded in paraffin, and sectioned (5 μm thickness) for subsequent staining. As per the manufacturer’s directive, sections were stained with anti-Ki67, anti-Active-Caspase-3, and anti-PSAT1 antibodies utilizing the MaxVision kit (Maixin, Fuzhou, China). Diaminobenzidine (0.05%) was the chosen chromogen, and hematoxylin served as the counterstain. Prepared sections were then mounted using Permount™ Mounting Medium and visualized under inverted fluorescence microscopy.

### Statistical analysis

All *in vitro* experiments were repeated thrice, with data expressed as mean ± standard deviation (SD). Comparisons between two groups were made employing a 2-tailed Student’s *t*-test. When analyzing variance across multiple groups, one-way ANOVA was employed, followed by *post-hoc* comparison using Tukey’s test. Statistical analyses were conducted utilizing the GraphPad Prism 8 software. A value of *p* < 0.05 was considered indicative of statistical significance.

## Results

### Cyclophosphamide combined with fludarabine chemotherapy five days later and then given CD19 CAR-T cells has the best effect in treating B-cell malignant tumors

**Figure 1:**
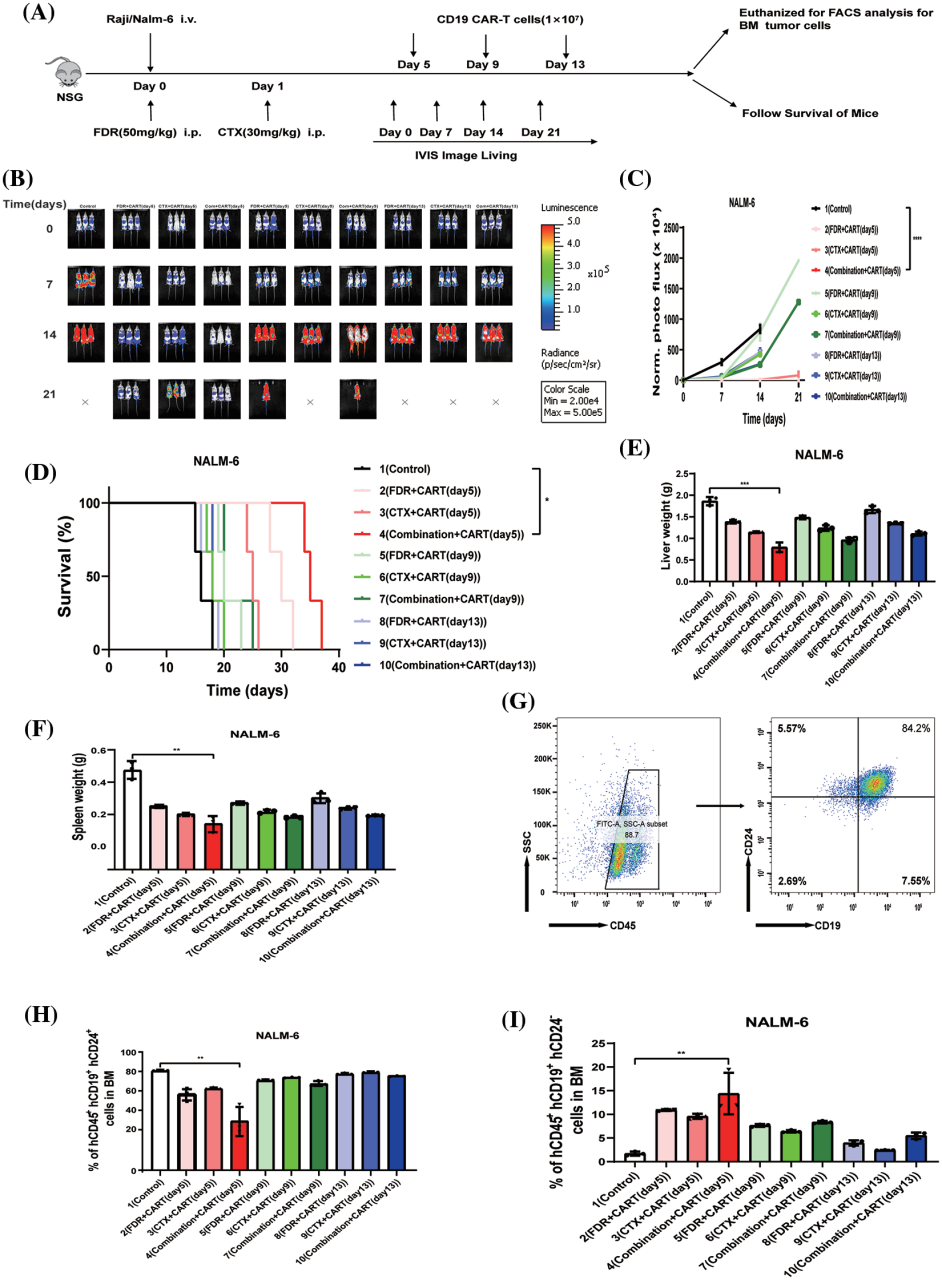
The best effect in treating B-ALL is to give CD19 CAR-T cells five days after cyclophosphamide combined with fludarabine chemotherapy. (A) Illustration of the B-cell malignancy xenograft model: NSG mice received intravenous injections of 5 × 10^5^ Nalm-6-luciferase (Nalm-6-luc) or 5 × 10^6^ Raji-luciferase (Raji-luc) on day 0. To determine the engraftment level and to randomize the treatment cohorts, bioluminescent imaging (BLI) was performed on day 0. Different groups received 1 × 10^7^ CD19 CAR-T cell treatments post various chemotherapeutic interventions: 5 days post FDR (50 mg/kg.i.p.), 5 days post CTX (30 mg/kg.i.p.), 5 days post FDR combined with CTX, 9 days post FDR, 9 days post CTX, 9 days post FDR combined with CTX, 13 days post FDR, 13 days post CTX, and 13 days post FDR combined with CTX. One group received PBS as a control. Serial BLI was conducted on days 7, 14, and 21. The BLI radiance quantification acted as a surrogate marker for B-cell malignancy tumor load. (B) BLI results prior to any treatment (day 0) and on days 7, 14, and 21, post Nalm-6-luc transplantation. (C) Progression of bioluminescent signals for each treatment cohort over time. Displayed data are mean values of each group ± standard deviation (SD). The results amalgamate data from three distinct experiments. (D) Kaplan-Meier survival analysis. Log-rank (Mantel-Cox) statistical tests were employed to assess survival differences among the cohorts. Data were collated from three individual experiments. (E) Synopsis of liver weights in mice, gauged 21 days post-leukemia transplantation. (F) Compilation of spleen weights in mice, measured 21 days subsequent to leukemia transplantation. (G) Exemplary flow cytometric evaluation of bone marrow, 21 days after the leukemia transplantation. The proportion of human CD45^^^ + CD19^^^ + CD24^^^+ Nalm-6 cells is denoted. (H) Overview of leukemic cell engraftment within the mouse bone marrow, determined 21 days post-leukemia transplant. The percentage of human CD45^^^ + CD19^^^ + CD24^^^ + Nalm-6 cells is specified. (I) Recapitulation of CD19 CAR-T cell engraftment in mouse bone marrow, assessed 21 days following leukemia transplantation. The percentage of human CD45^^^ + CD19^^^ + CD24^^^-CD19 CAR-T cells is highlighted. *p < 0.05; **p < 0.01; ***p < 0.001; ****p < 0.0001.

**Figure 2:**
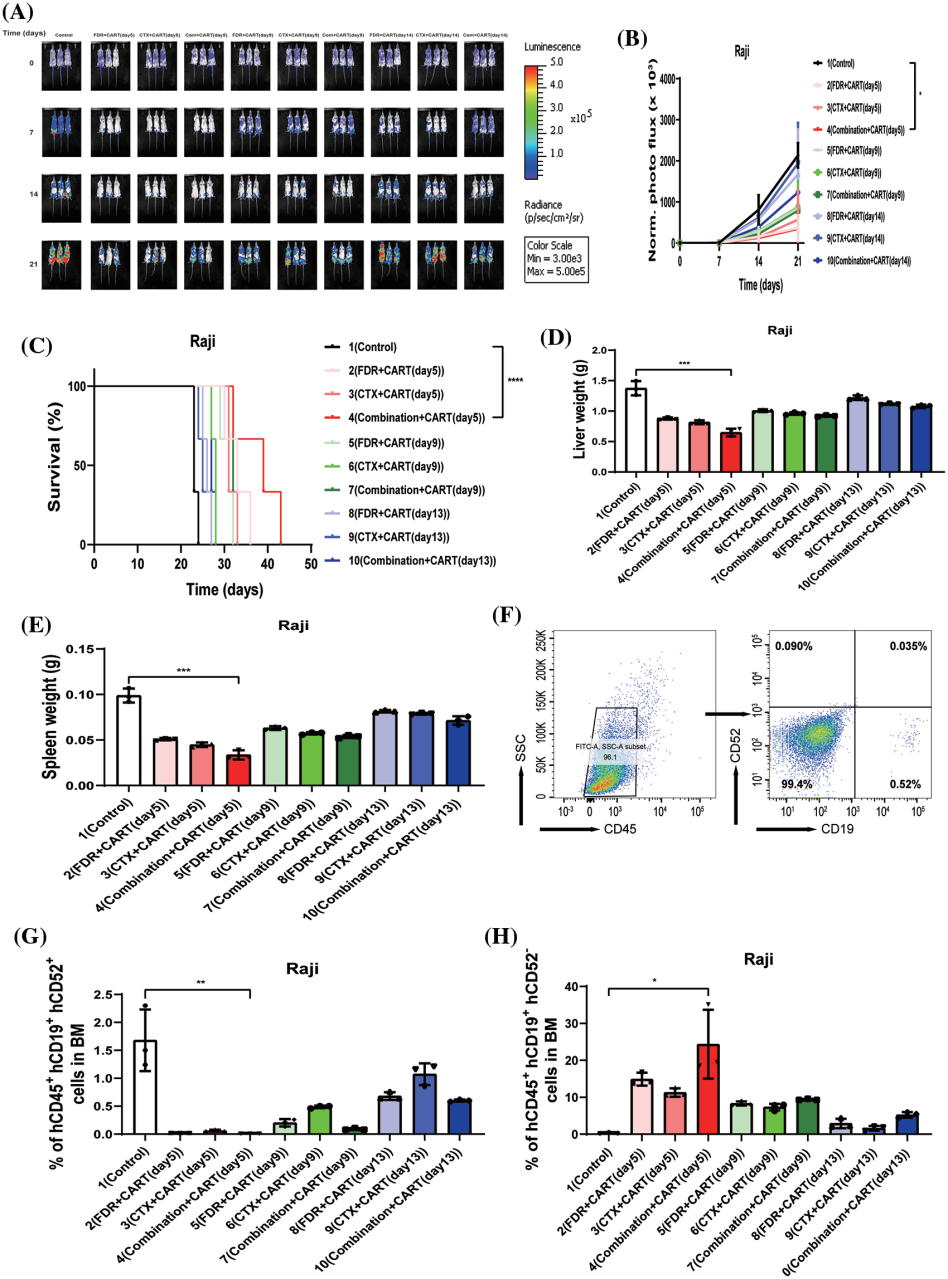
Therapeutic efficacy in Burkitt’s lymphoma mouse model. (A) BLI assessment pre-treatment (day 0) and subsequently on days 7, 14, and 21, post Raji-luc transplantation. (B) Chronological progression of bioluminescent signals across the treatment cohorts. The presented data encompass mean values for each group ± standard deviation (SD). The collated results derive from three discrete experiments. (C) Kaplan-Meier survival delineation. Statistical survival comparisons between cohorts were conducted using Log-rank (Mantel-Cox) tests. These results amalgamate data from three independent investigations. (D) Collation of liver weight measurements in mice, gauged 21 days post-lymphoma transplantation. (E) Compilation of spleen weight metrics in mice, assessed 21 days following lymphoma transplantation. (F) Exemplary flow cytometric assessment of bone marrow, 21 days subsequent to lymphoma transplantation. The indicated proportion pertains to human CD45^^^ + CD19^^^ + CD52^^^ + Raji cells. (G) Overview of lymphoma cell engraftment within the mouse bone marrow, determined 21 days post-lymphoma transplant. The denoted percentage represents human CD45^^^ + CD19^^^ + CD52^^^ + Raji cells. (H) Recapitulation of CD19 CAR-T cell engraftment in mouse bone marrow, measured 21 days post-lymphoma transplantation. The specified percentage pertains to human CD45^^^ + CD19^^^ + CD52^^^ − CD19 CAR-T cells. *p < 0.05; **p < 0.01; ***p < 0.001; ****p < 0.0001.

To scrutinize the therapeutic impacts of varied chemotherapy regimens in conjunction with CD19 CAR-T cells on B-cell malignancies, and to ascertain the optimal time interval for integrating CD19 CAR-T post-chemotherapy, we harnessed the Nalm-6 and Raji cell lines (which consistently express luciferase) and NSG mice to establish xenograft models of B-cell malignancies. Subsequent to the engraftment of 5 × 10^5^ Nalm-6-luc cells, we employed Bioluminescent Imaging (BLI) to validate the cellular implantation. A mere 24 h thereafter, cyclophosphamide and/or fludarabine were administered intraperitoneally as per predefined protocols. This was promptly succeeded by an infusion of 1 × 10^7^ CD19 CAR-T cells, with the subjects continually monitored utilizing BLI (Fig. 1A). Remarkably, in mice engrafted with B-ALL, an initial regimen of cyclophosphamide coupled with fludarabine, followed by CD19 CAR-T cell therapy on the fifth day, manifested superior efficacy in both diminishing tumor mass and enhancing survival rates (Figs. 1B–1D). This therapeutic regimen also displayed pronounced proficiency in ameliorating hepatic and splenic hypertrophy in B-ALL-afflicted mice (Figs. 1E and 1F). Concurrently, this specific protocol optimized the eradication of B-ALL cells within the murine bone marrow while concurrently minimizing the cytotoxic repercussions of chemotherapy on CD19 CAR-T cells (Figs. 1G–1I). Conclusively, the administration of CD19 CAR-T cell therapy five days post cyclophosphamide and fludarabine preconditioning emerged as the most potent therapeutic strategy against B-ALL. Analogous therapeutic outcomes were discerned in the Burkitt’s lymphoma mouse model (Fig. 2).

### Cyclophosphamide combined with fludarabine chemotherapy and CD19 CAR-T cells given five days later to treat B-cell malignant tumors may work by interfering with cell metabolism

**Figure 3:**
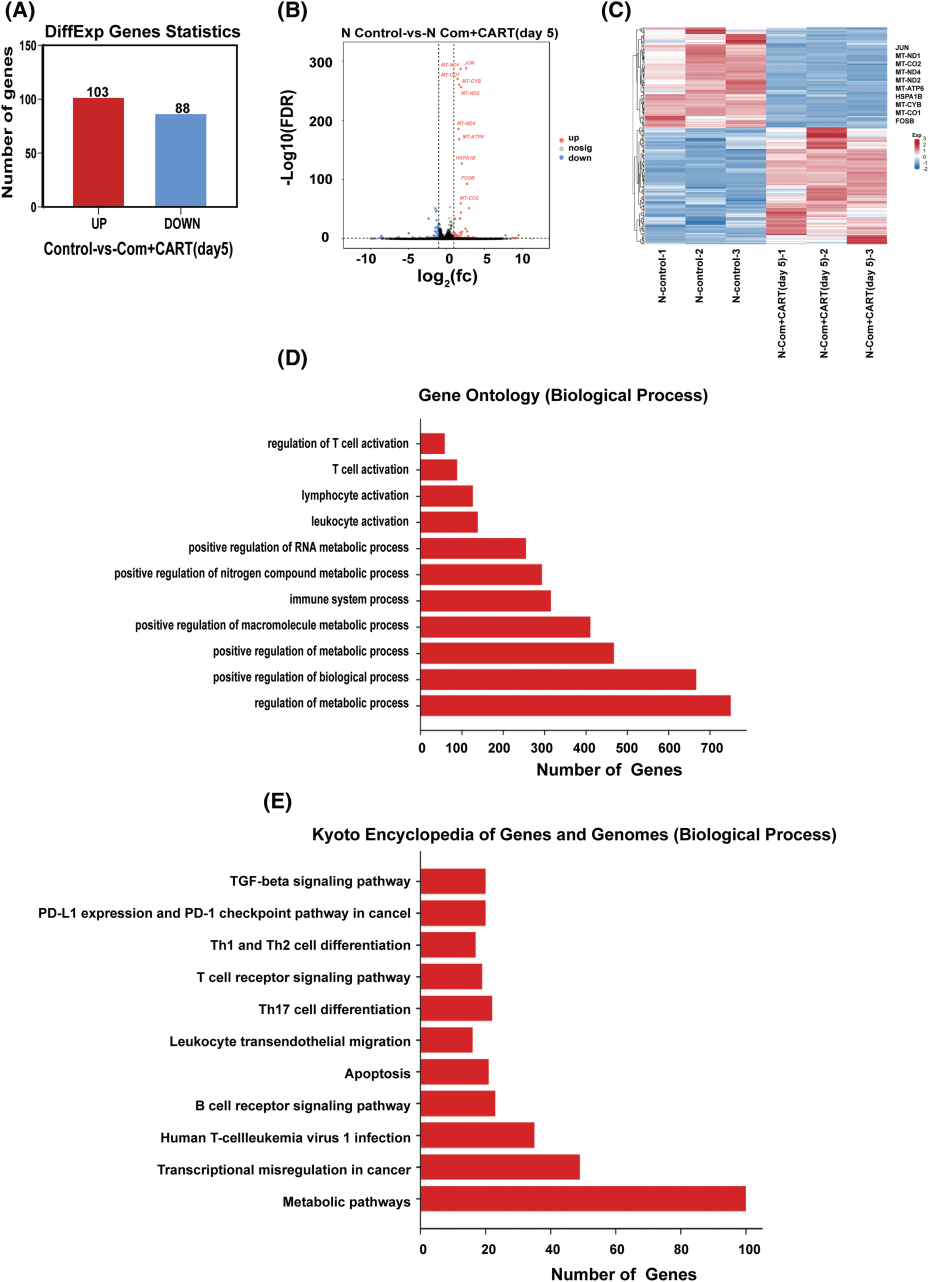
RNA sequencing results indicate that this approach mainly works by interfering with the metabolism of B-ALL cells. (A–C). Through RNA-seq analysis, differentially expressed genes (DEGs) were discerned between samples of the control and the treated cohorts. The quantitative delineation (A) and a visually articulated volcano plot (B) of DEGs are showcased. For a DEG to be considered significant, the criteria were set at FDR < 0.05 and |log_2_ FC| > 1. A heatmap elucidates the top 10 ascendant or descendant genes when comparing lycorine with control (C). (D) The Gene Ontology (GO) pathway enrichment analysis pertaining to DEGs is delineated. X-axis represents the number of DEGs in the analogous pathway, whereas the Y-axis showcases functional pathways. (E) The Kyoto Encyclopedia of Genes and Genomes (KEGG) pathway enrichment analysis concerning DEGs is illustrated. The X-axis details the number of DEGs within a given pathway, with the Y-axis presenting the corresponding functional pathways.

**Figure 4:**
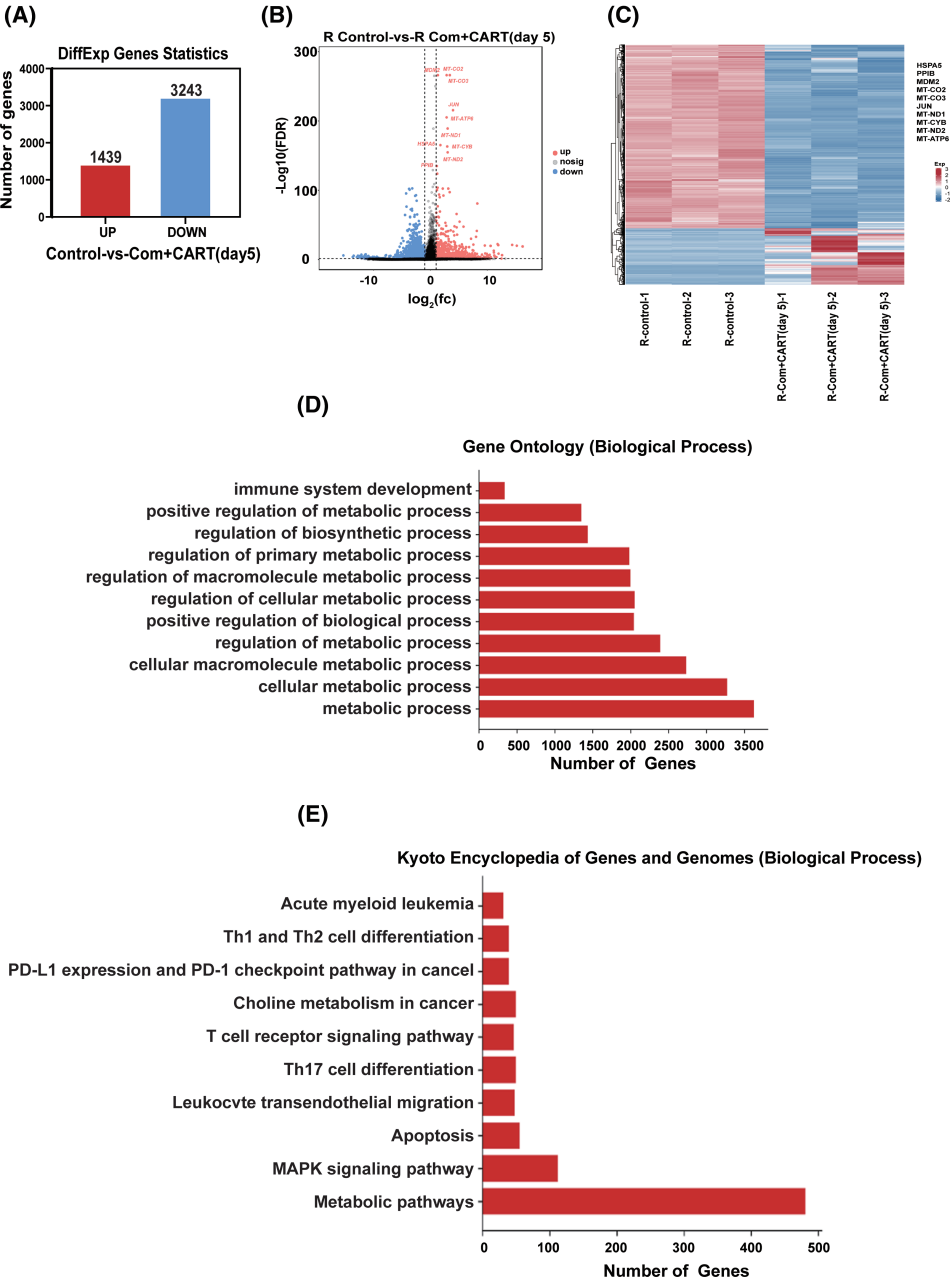
RNA sequencing results indicate that this approach mainly works by interfering with the metabolism of Burkitt’s lymphoma cells. (A–C) RNA-seq analysis enabled the discernment of differentially expressed genes (DEGs) between samples derived from control and treated cohorts. A quantitative representation (A) alongside a visualized volcano plot (B) characterizes these DEGs. An established threshold for significance was set at FDR < 0.05 and |log_2_ FC| > 1. A heatmap provides a vivid display of the top 10 either upregulated or downregulated genes when juxtaposing lycorine to control (C). (D) The Gene Ontology (GO) pathway enrichment analysis relating to DEGs is meticulously delineated. The X-axis specifies the number of DEGs within an analogous pathway, while the Y-axis demarcates distinct functional pathways. (E) A detailed representation of the Kyoto Encyclopedia of Genes and Genomes (KEGG) pathway enrichment analysis for DEGs is provided. On the X-axis, the enumeration of DEGs in the corresponding pathway is highlighted, whereas the Y-axis defines the associated functional pathways.

**Figure 5:**
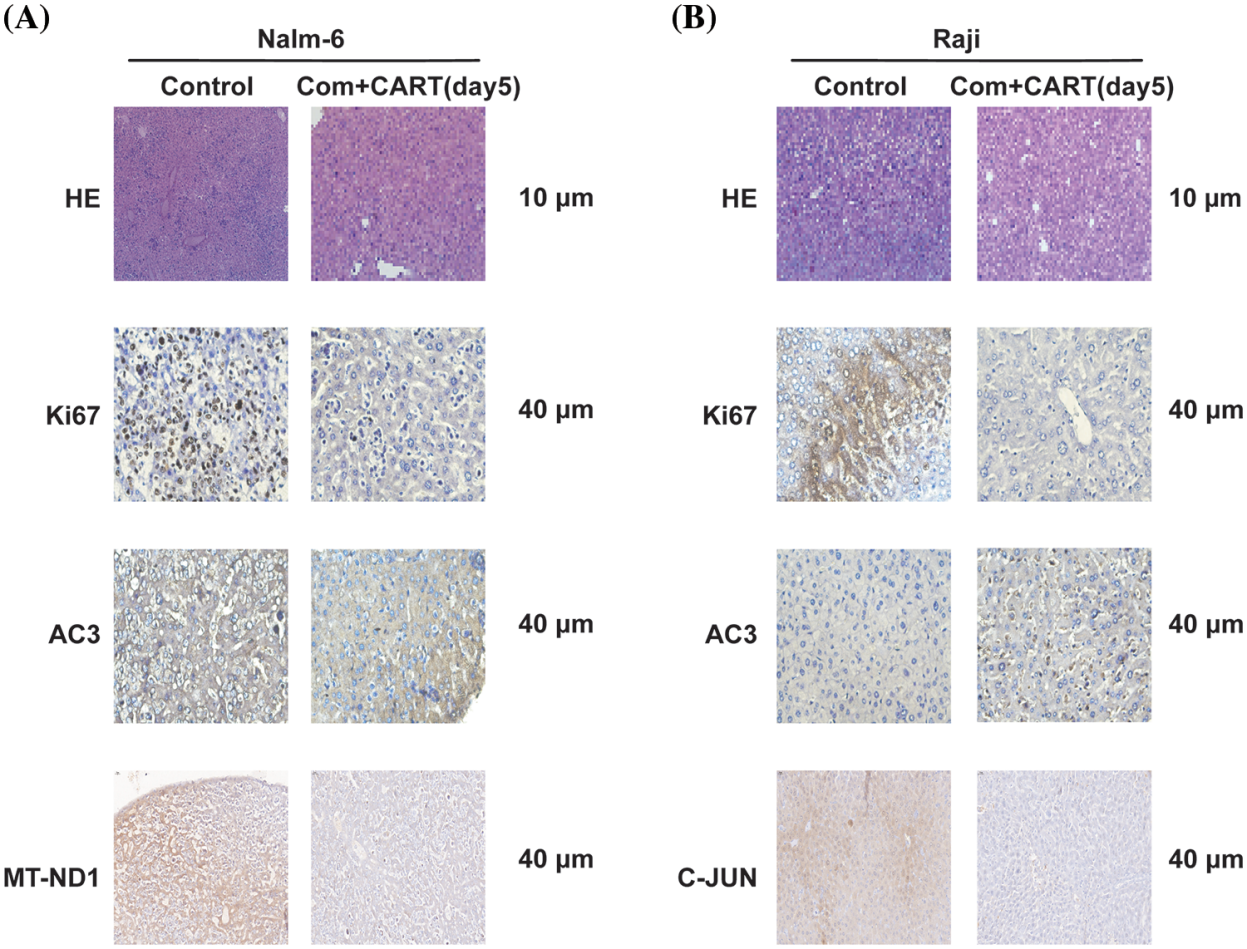
Histological examination of liver sections from murine cohorts at day 21 via H&E and IHC staining. Stainings with antibodies against Ki67, AC3, and MT-ND1 (A) or C-JUN (B) are showcased. Images were captured at an original magnification of 40 times.

To elucidate the underpinning mechanism by which this therapeutic strategy impacts B-cell malignancies *in vivo*, we proceeded to the experimental culmination. Subsequent to the experiment, mice from both the treatment and control cohorts were euthanized. Post mortem, we isolated PBMCs and executed RNA sequencing. Intriguingly, in the B-ALL murine model, the treatment cohort displayed a significant modulation of B-ALL cell metabolism when juxtaposed with the control. Specifically, the regimen appeared to attenuate the expression of a repertoire of genes implicated in mitochondrial metabolism, exemplified by JUN (Fig. 3). Parallel findings were gleaned from the Burkitt’s lymphoma murine model (Fig. 4). Complementarily, histological examinations encompassing Hematoxylin and Eosin (HE) staining, alongside immunohistochemical analyses of the hepatic tissues from both cohorts, were undertaken. These analyses evinced that, relative to the control cohort, the treatment regimen markedly diminished the hepatic infiltration of neoplastic cells and stifled their proliferation. Concurrently, a pronounced downregulation of mitochondrial-associated genes, notably JUN and MT-ND1, was observed (Fig. 5). Collectively, these findings suggest that the amalgamation of cyclophosphamide and fludarabine chemotherapy, succeeded by the introduction of CD19 CAR-T cells after a five-day interval, potentially exerts its antineoplastic efficacy by perturbing the metabolic pathways within tumor cells.

## Discussion

Owing to its cytotoxic properties, chemotherapy does not solely target neoplastic cells and deplete the endogenous T cell cohort within the bone marrow; it also potentially impairs the functional capacity and proliferative prowess of CAR-T cells circulating in the bloodstream. Administering low-dose induction chemotherapy prior to CD19 CAR-T cell therapy has been discerned to significantly ameliorate the clinical outcomes for oncology patients. Nevertheless, the optimal chemotherapy schema and the precise timing between chemotherapy and the administration of CD19 CAR-T therapy remain paramount in prognostic considerations. The combination of cyclophosphamide and fludarabine stands as a predominant preparatory regimen preceding CAR-T cell therapy. A pivotal clinical trial orchestrated by the American Institute for Disease Control and Prevention, which encompassed 53 pediatric and adolescent participants diagnosed with R/R ALL, assessed the differential efficacies of chemotherapy regimens relative to tumor burden. Notably, modulating the chemotherapy based on the disease burden did not confer additional therapeutic advantages, with overall survival being conspicuously superior in cohorts receiving the fludarabine/cyclophosphamide duo compared to alternative regimens [14]. In the context of relapsed or refractory B-ALL, the lymphodepletion strategy entailing the concomitant use of cyclophosphamide and fludarabine has manifested a staggering 94% complete remission rate prior to CD19 CAR-T cell therapy, accompanied by a restructured Disease-free survival (DFS) that surpassed that of the fludarabine-excluded group [15]. In advanced lymphoma cases, subsequent to a low-dose chemotherapy induction employing cyclophosphamide and fludarabine, CD19 CAR-T therapy administered after a 48-h interval led to an overall patient response rate of 73%. However, this was counterbalanced by 55% of patients manifesting grade 3 or 4 neurotoxic adverse events [16]. Currently, most patients with aggressive non-Hodgkin lymphoma (NHL) will undergo induction with R-CHOP (rituximab, cyclophosphamide, doxorubicin, vincristine, and prednisone) or a similar regimen. Meanwhile, the FDA has approved commercial CAR T-cell products axicabtagene ciloleucel (axi-Cel; Yescarta Kite/Gilead), tisagenlecleucel (tisa-cel; Kymriah Novartis) and lisocabtagene maraleucel (liso-cel; Breyanzi Juno BMS) for relapsed or clinical treatment of refractory (R/R) aggressive B-cell lymphoma. Thus, the judicious amalgamation of chemotherapy and CAR-T therapy warrants meticulous evaluation. Our experimental data further underscores that a treatment regimen involving CD19 CAR-T therapy postulated with cyclophosphamide and fludarabine outperforms either chemotherapy agent in isolation, irrespective of whether CD19 CAR-T therapy is introduced on day five, nine, or thirteen post-chemotherapy.

Preliminary investigations in this domain authenticated the modulatory impact of antecedent chemotherapy on CD19 CAR-T cellular dynamics via *in vitro* assessments. Findings indicated that both fludarabine and mafosfamide might attenuate CD19 CAR-T cell functionality, culminating in programmed cell death [17]. The durability of CAR-T cells in the host is recognized as a cardinal determinant of CAR-T therapeutic success [18]. Preceding chemotherapy with CAR-T therapy can obliterate lymphocytes, pivotal for the rapid post-infusion expansion and proliferation of patient-derived CAR-T cells. Presently, a consensual clinical blueprint detailing the interval between chemotherapy and CAR-T cell therapy remains elusive. In this investigation, through rigorous *in vivo* preclinical evaluations, it was discerned that in B-ALL and Burkitt’s lymphoma murine models, post lymphocyte depletion via cyclophosphamide and fludarabine, administering CD19 CAR-T therapy on day five yielded optimal neoplastic cell abrogation and sustained CD19 CAR-T cell vitality, thereby optimizing murine survival trajectories.

Mitochondria, the cellular powerhouses, not only furnish the fundamental substrates requisite for neoplastic metabolism but also modulate redox and calcium equilibria, partake in transcriptional oversight, and orchestrate cellular apoptosis [19], Intriguingly, neoplastic cells exhibit a distinct metabolic predilection, marked by an augmented reliance on aerobic glycolysis, distinguishing them from their non-neoplastic counterparts–a phenomenon that stands as a defining hallmark of cancer [20]. Contemporary therapeutic avenues, strategically targeting aberrant mitochondrial metabolism within cancer cells, are progressively gaining traction in the oncological landscape [21]. The current investigation delineates that, relative to the control cohort, the administration of CD19 CAR-T cell therapy, five days subsequent to a combined regimen of cyclophosphamide and fludarabine, markedly disrupts the metabolic pathways in murine models of B-ALL and Burkitt’s lymphoma. Notably, this therapeutic perturbation is postulated to be achieved via the modulation of specific mitochondrial targets within the tumor cells.

This research provides compelling evidence, derived from rigorous preclinical *in vivo* assessments, supporting the superior efficacy of a sequential regimen–cyclophosphamide and fludarabine chemotherapy followed by CD19 CAR-T cell therapy–compared to either chemotherapeutic agent alone in targeting B-cell malignancies. The most pronounced anti-neoplastic effect was observed when CD19 CAR-T cells were administered five days after combined chemotherapy. Preliminary findings intimate that this pronounced effect is intricately linked to the disruption of mitochondrial metabolic processes within the tumor cells. While these findings undoubtedly bolster the potential of this therapeutic strategy, a more expansive repertoire of preclinical and clinical studies remains imperative for definitive validation.

## Data Availability

All data are incorporated into the article and will be made available on request.

## List of Abbreviations

CAR-T: Chimeric antigen receptor T
CTX: Cyclophosphamide
FDR: Fludarabine
BTK: Bruton’s tyrosine kinase
TCR: T-cell receptor
HLA: Human leukocyte antigen
BLI: Bioluminescent imaging
scFv: Single-chain variable fragment
